# The Regenerative Effect of Bone Marrow-Derived Stem Cells on Cell Count and Survival in Acute Radiation Syndrome

**Published:** 2017-01

**Authors:** Seyed Mahmood Reza Aghamir, Davood Mehrabani, Masoud Amini, Mohammad Amin Mosleh-Shirazi, Samaneh Nematolahi, Fatemeh Shekoohi-Shooli, Seyed Mohammad Javad Mortazavi, Iman Razeghian Jahromi

**Affiliations:** 1 Department of Radiology and Radiotherapy, School of paramedical, Shahid Beheshti University of Medical Science, Tehran, Iran;; 2 Stem Cell and Transgenic Technology Research Center, Shiraz University of Medical Sciences, Shiraz, Iran;; 3 Department of Surgery, School of Medicine, Shiraz University of Medical Sciences, Shiraz, Iran;; 4 Department of Radiotherapy, Nemazee Hospital, Shiraz University of Medical Sciences, Shiraz, Iran;; 5 Department of Biostatistics, School of Medicine, Shiraz University of Medical Sciences, Shiraz, Iran;; 6 Ionizing and Non-Ionizing Radiation Protection Research Center, Shiraz University of Medical Sciences, Shiraz, Iran

**Keywords:** Acute radiation syndrome, Mesenchymal stem cells, Bone marrow, Survival, Cell count

**DEAR EDITOR**

Acute radiation syndrome (ARS) is called as radiation sickness or radiation toxicity caused by abnormally high exposure to ionizing radiation in a very short period of time.^1^ High doses of ionizing radiation are able to contribute to detrimental systemic effects in different organs.^2^ In treatment of patients with ARS, physicians have used growth factors, cytokines and bone marrow transplantation.^3^ Mesenchymal stem cells (MSCs) have the potential for multilineage differentiation.^4^^,^^5^ Bone marrow-derived stem cells (BMSCs) are the most well- known type of the mesenchymal stem cells used with safety and efficacy in several diseases such as ARS.^1^ The present study assessed the regenerative effect of bone marrow-derived stem cells on cell count and survival in Acute Radiation Syndrome.

For MSC culture, both femoral and tibial bones from male mice were removed and after removal of muscular and connective tissues, the bones were cut at both ends and the bone marrow was flushed out into a 15 ml falcon tube filled with Dulbecco’s Modified Eagle Medium (DMEM; Biovet, Bulgaria) and 1% penicillin streptomycin (Sigma, USA) and centrifuged at 1200 rpm for 5 minutes. The precipitate was cultured in 25 cm^2^ flasks containing DMEM supplemented with 10% fetal bovine serum (FBS; Biovet, Bulgaria), 1% L-glutamine (Sigma, USA) and 1% penicillin and streptomycin. The culture flasks were transferred into CO2 incubator while the medium was changed every 3 days. 

The adherent cells were subcultured until passage 5 while they were counted for survival rate. The osteogenic was evaluated with Alizarin Red staining (Sigma, USA). RT-PCR was conducted to evaluate the expression of MSC markers. Forty 8-12 weeks male mice were randomly divided into 2 equal groups. Group A received no BMSCs but group B underwent 150×10^3^ cells of passage 5 in 150 µl medium of BMSC transplantation intravenously into the tail, 24 hours after γ irradiation. Both groups were irradiated with 10 Gy (dose rate .286 Gy/ min) ^60^CO, during 35 minutes with a field size of 35×35 for all the body area. 

**Fig. 1:**
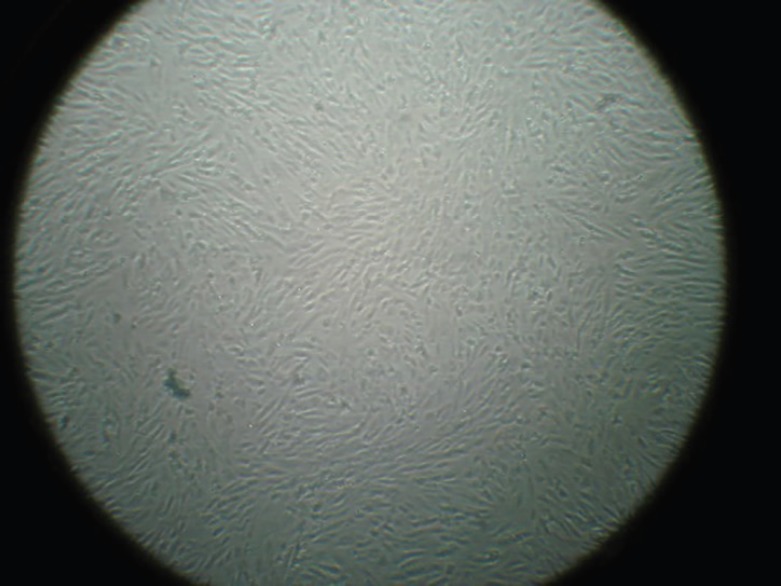
Bone marrow-derived stem cells in 3^rd^ passage

**Fig. 2:**
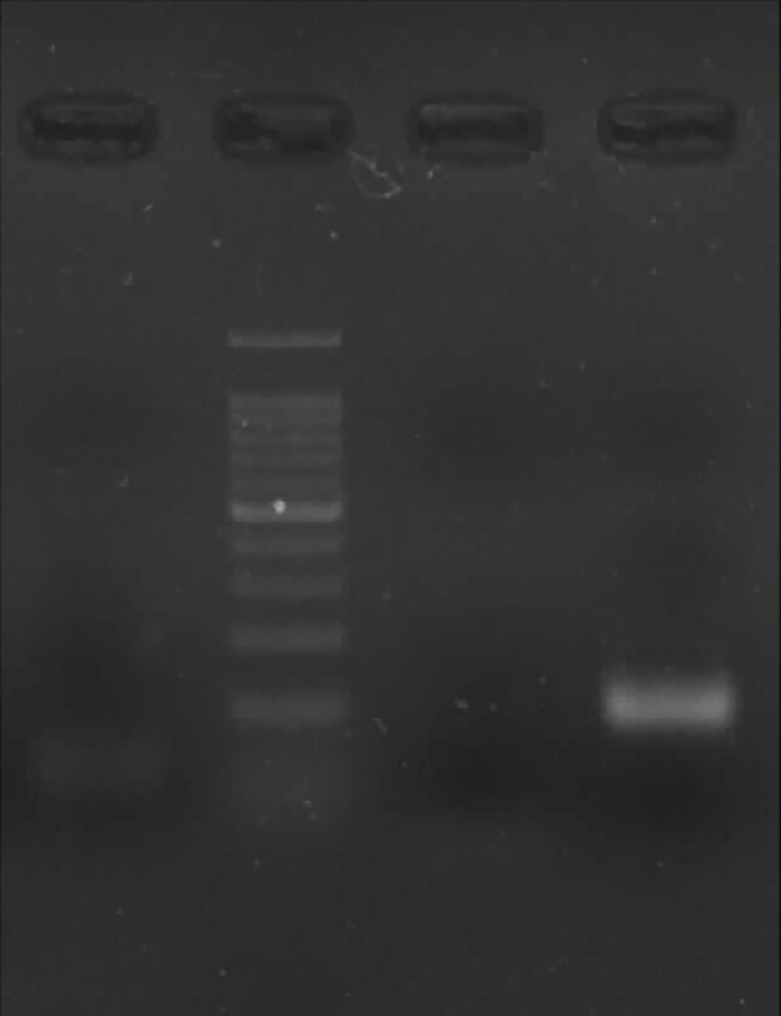
RT-PCR with positive expression of CD90 and absence of CD34 and CD45 (Ladder, CD90, CD34, 3: CD45

**Fig. 3:**
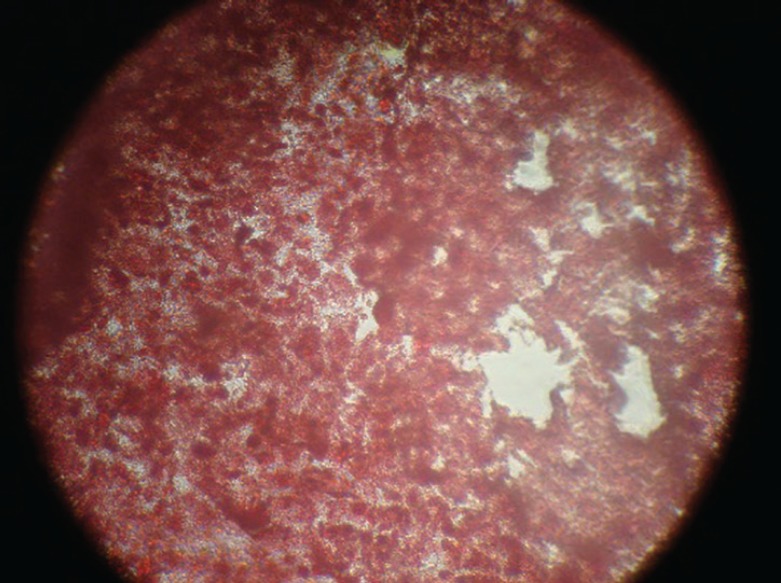
Alizarin red staining with osteogenic differentiation of bone marrow-derived stem cells

BMSCs were plastic adherent and spindle-shape (Figure 1) and expressed CD90 marker but not CD34 and CD45 (Figure 2). Culture of BMSCs in osteogenic media lead to osteogenic differentiation of these cells (Figure 3). A significant increase was noticed for the number cells in bone marrow in group B when compared to group A, one week after γ irradiation (*p*=0.0001, Table 1). The mortality rate one and two weeks after γ irradiation was demonstrated in Table 2.

**Table 1:** Cell count in bone marrow one week after γ irradiation

**Variable**	**No.**	**Cell count of bone marrow** **(Mean±SD)**
Group A (no receive BM-MSCs)	6	1.77×10^7^±20×10^6^
Group B (receive BM-MSCs)	6	2.47×10^7^±2×10^6^
*p* value		0.0001

**Table 2:** Mortality rate one and two weeks after γ irradiation

**Group**	**Time (day)**	**No.**	**Status**
A	1 3 4 8 9 10	2 1 2 2 3 2	Dead Dead Dead Dead Dead Dead
B	1 3 11	2 1 1	Dead Dead Dead

BMSCs were shown to differentiate into various cells, and secrete cytokine and growth factors, and have immunomodulatory properties through paracrine and endocrine mechanisms in injured tissue.^6^ Eaton *et al.* showed that MSC therapy can be effective for acute radiation syndrome due to the fact that MSCs have immunomodulatory properties.^7^ Guo *et al.* in a 32-year-old man who was exposed to whole body dose of 14.5 GY γ- radiation concluded that cell therapy was an effective approach and significantly decreased mortality rate.^8^ Lange *et al.* found that systemic administration of MSCs had healing effects in ARS and managed radiation.^3^ Chapel *et al.* revealed that MSCs can migrate to the site of injury and repair the injured tissue.^9^ The therapeutic use of compact of BMSCs was shown to reduce the injury and increase the survival rate after lethal whole body irradiation.^10^^,^^11^

These studies confirm our findings demonstrating that BMSCs reduced the detrimental effects of radiation and increased the survival rate in ARS. Based on our findings, BMSCs can be recommended in reduction of detrimental effects of ARS and decreasing the mortality after exposure to γ irradiation.
